# High infection rates for onchocerciasis and soil-transmitted helminthiasis in children under five not receiving preventive chemotherapy: a bottleneck to elimination

**DOI:** 10.1186/s40249-022-00973-1

**Published:** 2022-04-28

**Authors:** Hugues C. Nana-Djeunga, Linda Djune-Yemeli, André Domche, Cyrille Donfo-Azafack, Arnauld Efon-Ekangouo, Cédric Lenou-Nanga, Narcisse Nzune-Toche, Yves Aubin Balog, Jean Gabin Bopda, Stève Mbickmen-Tchana, Velavan P. Thirumalaisamy, Véronique Penlap-Beng, Francine Ntoumi, Joseph Kamgno

**Affiliations:** 1 Centre for Research on Filariasis and other Tropical Diseases (CRFilMT), Yaoundé, Cameroon; 2 Molecular Diagnosis Research Group, Biotechnology Centre, University of Yaoundé I, Yaoundé, Cameroon; 3 Department of Biochemistry, Faculty of Science, University of Yaoundé I, Yaoundé, Cameroon; 4 Parasitology and Ecology Laboratory, Department of Animal Biology and Physiology, Faculty of Sciences, University of Yaoundé I, Yaoundé, Cameroon; 5 Institute for Tropical Medicine, University of Tübingen, Tübingen, Germany; 6 Fondation Congolaise pour la Recherche Médicale (FCRM), CG-BZV, Brazzaville, Republic of the Congo; 7 Faculty of Science and Technology, Marien Ngouabi University, Brazzaville, Republic of the Congo; 8 Department of Public Health, Faculty of Medicine and Biomedical Sciences, University of Yaoundé I, Yaoundé, Cameroon

**Keywords:** Onchocerciasis, Soil-transmitted helminthiasis, Preventive chemotherapy, Children under five, Cameroon

## Abstract

**Background:**

The current mainstay for control/elimination of onchocerciasis and soil-transmitted helminthiasis (STH) relies on ivermectin- and mebendazole/albendazole-based preventive chemotherapies. However, children under five years of age have been excluded in both research activities and control programs, because they were believed to have insignificant infection rates. There is therefore a need for up-to-date knowledge on the prevalence and intensity of STH and onchocerciasis infections in this age group. This study aimed at assessing the rates and intensities of onchocerciasis and STH infections in children under five years of age who are excluded from ivermectin- or mebendazole/albendazole-based preventive chemotherapies.

**Methods:**

A series of cross-sectional surveys was conducted in four Health Districts in the Centre and Littoral Regions of Cameroon between 2018 and 2019. All subjects aged 2 to 4 years, were screened for prevalence (or infection rate) and intensity [number of eggs per gram of stool (epg) or number of microfilariae per skin snip (mf/ss)] of STH and onchocerciasis infections respectively using the Kato-Katz and skin snip methodologies. Chi-square and the non-parametric tests (Mann Whitney and Kruskal Wallis) were used to compare infection rates and intensities of infections between Health Districts and genders, respectively.

**Results:**

A total of 421 children were enrolled in this study. The overall prevalence of onchocerciasis was 6.6% [95% confidence interval (*CI*): 4.3‒9.9], ranging from 3.6% (in the Ntui Health District) to 12.2% (in the Bafia Health District). The intensity of infection ranged from 0.5 to 46 microfilariae per skin snip [median: 5; interquartile range (IQR): 2.25‒8.5]. The overall prevalence of STH was 9.6% (95% *CI*: 6.5‒13.9), with a high infection rate (29.6%) in the Akonolinga Health District. Two STH species (*Ascaris lumbricoides and Trichuris trichiura*) were found among infected individuals. The median intensities of STH infections were 1,992 epg (IQR: 210‒28,704) and 96 epg (IQR: 48‒168) for *A. lumbricoides* and *T. trichiura,* respectively.

**Conclusions:**

This study reveals that children < 5 years of age are highly infected with STH and onchocerciasis, and could contribute to the spread of these diseases, perpetuating a vicious circle of transmission and hampering elimination efforts. These findings reveal the urgent need to provide (or scale) treatments (likely pediatric formulations) to these preschool-aged children, especially in areas of high transmission, to accelerate efforts to reach WHO 2030 target.

**Graphical Abstract:**

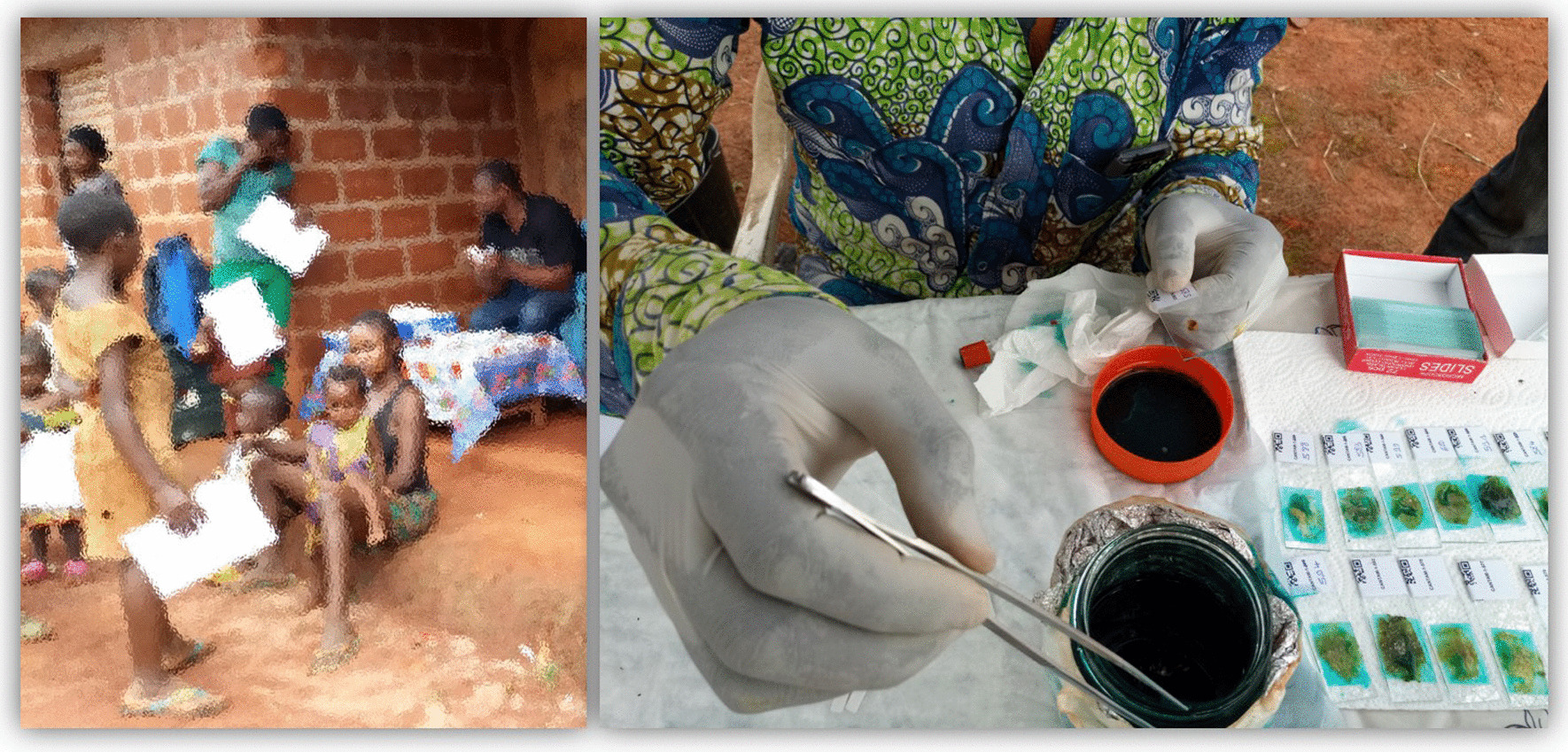

## Background

In 2001, the World Health Organization (WHO) recommended annual or bi-annual administration of a single dose albendazole (ALB, 400 mg) or mebendazole (MEB, 500 mg) to STH high-risk populations. These include preschool-age children (pre-SAC), school-age children (SAC), women of childbearing age and adults who are particularly exposed to the infection in endemic areas, such as tea pickers [1]. Due to logistical constraints, only SACs were treated regularly [2], while the other groups being neglected. As the need for preventive chemotherapy (PC) for other at-risk groups became increasingly clear [1, 3], WHO reformulated guidelines and recommended new targets for STH control, including 100% of endemic countries achieving 75% coverage in the pre-SAC and SAC. Despite this new recommendation, pre-SAC phase lagged behind, because the infection rate in this age group was considered insignificant [4, 5]. Consequently, only 15% of endemic countries reported consistently administering PC to pre-SAC for more than 5 years [6], so additional epidemiological evidence of STH in these children is urgently needed to intensify control measures in this age group.

The control/elimination strategy for onchocerciasis mainly relies on ivermectin (IVM)-based PC, the so-called community-directed treatment with ivermectin (CDTI). Although IVM is distributed based on heigh or weight of individuals aged ≥ 5 years in endemic communities [7, 8]; children less than 5 years are systematically excluded from PC [9, 10], probably for practical reasons and because of the lack of epidemiological evidence in this age group. Indeed, early investigations indicated that children under five are very unlikely to harbor *Onchocerca volvulus* microfilariae [11, 12], and no safety and efficacy trials had been conducted for this age class [13]. As a consequence, children who are exposed to a similar risk of infection—and probably even infected—as their older peers (but not receiving PC), have an increased risk of developing nodding syndrome or onchocerciasis-associated epilepsy (OAE) as they grow older, as previously demonstrated [14–17]. Importantly, these untreated children might also constitute a human reservoir for the parasite, preventing the prospect of elimination through continued transmission. Therefore, there is a need to accumulate more evidence and update epidemiological data on onchocerciasis in children under five years of age for subsequent actions.

The WHO 2030 targets for onchocerciasis and soil-transmitted helminthiases (STH) are to interrupt the transmission in 31% of endemic countries, and to achieve and/or maintain the elimination as public health concern [6, 18, 19]. However, to reach these ambitious goals, control/elimination strategies for the helminthic diseases need to be extended to all age groups, based on appropriate evidence. We therefore conducted a series of cross-sectional studies in areas of varying endemicity for STH and onchocerciasis in Cameroon to determine the rates and intensities of onchocerciasis and STH infections in children under five years of age who are excluded from IVM- or MEB/ALB- based PC.

## Methods

### Study sites and population

This was a multicentric observational study conducted between 2018 and 2019 in four Health Districts (Akonolinga, Bafia, and Ntui in the Center Region, and Yabassi in the Littoral Region) in Cameroon, with varying levels of endemicity for onchocerciasis and STH.

The Akonolinga Health District, located in the Nyong-et-Mfoumou Division (Centre Region, Cameroon), is a forest zone with a plateau landscape. Its climate is of equatorial type with four seasons [20]. Estimated at 83,464 inhabitants [21], the populations are typically rural and their main activities are farming (coffee, cacao and food crops) and fishing. The area is highly endemic to STH and loiasis [22, 23], but hypo-endemic to onchocerciasis [24]. Therefore, IVM-based MDA is not implemented in the area, while school-based deworming with MEB has been introduced and implemented in the area since 2007.

The Bafia Health District, situated in the Mbam-et-Inoubou Division (Central Region, Cameroon), is a forest-savanna transition zone. The area is irrigated by many fast-flowing rivers (Sanaga, Mbam, Noun and their tributaries). The main activities of its 161,400 inhabitants are farming, fishing and sand mining [21]. Onchocerciasis is meso-endemic in the area, despite mass distribution of IVM ongoing for more than two decades [25].

The Ntui Health District is located in the Mbam-et-Kim Division (Centre Region, Cameroon), where a humid equatorial climate with four seasons prevails. In this forest-savanna transition zone, most of the tributaries of Mbam and Sanaga are surrounded by forest galleries. Due to its endemicity to onchocerciasis, CDTI was initiated in this area in 1999‒2000. In the Ntui Health District, a baseline survey conducted in the Mbangassina (one of the 11 Health Areas of the Ntui Health District), revealed high prevalence of STH (51% for *Ascaris lumbricoides* and 64% for *Trichuris trichiura*) (Kamgno et al. unpublished data).

Yabassi Health District in Nkam Division (Littoral Region, Cameroon) is a densely forested area with an equatorial climate with two seasons. The 652,683 inhabitants are mostly farmers. The area was known to be highly endemic for onchocerciasis and lymphatic filariasis, and community-based MDA with ALB and IVM has been implemented since 2000. This area was also known to be highly endemic to STH [26, 27]. School-based deworming with MEB is ongoing in the area since 2007 and a recent survey showed that STH transmission was almost interrupted, likely due to the combined effect of IVM- and MEB-based MDA [28].

Based on the availability of baseline data in the targeted settings, the Mbangassina Health Area (Ntui Health District) was surveyed for both STH and onchocerciasis, while Akonolinga Health District was screened only for STH, and Bafia and Yabassi Health Districts surveyed only for onchocerciasis.

### Study design

A series of four cross-sectional studies were undertaken to determine the magnitude (prevalence and intensity) of STH and onchocerciasis infections in children aged 2‒4 years. Although the enrollees were not expected to be involved in PC (either with ALB, MEB or IVM), the surveys took place approximately 11 months after last MDA campaign (either with IVM or MEB/ALB), to limit the potential impact of the latest treatments on the transmission of targeted infections. All individuals aged 2 to 4 years and whose parents consented for their participation were included in the study; their socio-demographic data (including the age, sex village of residence, number of years spent in the village of residence, or other villages where the participant lived before arriving in the village …) were recorded and they underwent parasitological (skin snip and Kato-Katz) and clinical examinations.

### Sample collection and processing

A 60 ml plastic screw-cap vial was given to all the eligible participants for stool collection. Stool samples were examined for the presence of eggs using the standard Kato-Katz technique [29]. Briefly, a single thick smear using a 41.7 mg template was prepared for each individual. The preparation was examined by qualified laboratory technicians, within one hour after slides’ preparation to ensure that all STH species present in the preparation should be identified. All STH eggs found on the slides were identified and counted, and the results expressed as eggs per gram of stool (epg). Based on the WHO criteria, STH infections were classified as light, moderate and heavy, for different parasite species, as follow: *A. lumbricoides* (light: 0–4,999 epg; moderate: 5000–49,999 epg; and heavy: ≥ 50,000 epg) and *T. trichiura* (light: 0–999 epg; moderate: 1000–9,999 epg; and heavy: ≥ 10,000 epg) [30].

Onchocerciasis was diagnosed by the skin snip technique. Indeed, two skin biopsies were taken from each posterior iliac crest using a 2 mm corneoscleral punch (Holth-type). The skin samples were immediately placed separately into wells of microtiter plates containing a sterile normal saline solution and incubated at room temperature [31]. The skin biopsies were examined microscopically for *O. volvulus* microfilariae (mf). In brief, after 24 h incubation at room temperature, the fluid from each well was examined under low magnification (× 40) by trained laboratory technicians. For positive results, the microfilariae were counted and individual microfilarial densities were expressed as the arithmetic mean number of microfilariae in the two skin snips.

### Statistical analyses

Microsoft Office Access was used to record field and laboratory data. The database was then exported to Microsoft Office Excel (Microsoft Co., Redmond, Washington, USA), formatted and cleaned before being definitively archived in Excel format. Statistical analyses were performed using GraphPad prism version 6 (GraphPad Software Inc., San Diego, CA, USA). Prevalence or infection rates were expressed as percentages with 95% confidence interval (*CI*) while intensities of infections were expressed as median of mf or eggs/larvae positive counts with interquartile range (IQR). Chi-square test was used to compare prevalence or infection rates between Health Districts and genders. The non-parametric Kruskal Wallis and Mann Whitney tests were used to compare intensities of infections between the different Health Districts and genders, respectively. The threshold for significance was set at 5% for all analyses.

## Results

### Socio-demographic characteristics of the study population

A total of 421 children aged 2 to 4 years (Median: 3; IQR: 2–4) were enrolled in the four Health Districts visited during this study. Most of the participants were originating from the Ntui Health District (40.1%); the sex ratio (M/F) was 0.99, with 50.4% of the participants being females (Table 1).

**Table 1:** Socio-demographic data of participants in the targeted Health Districts

Variables	Gender		Age range	Total
	Male	Female	(Median; IQR)	
Akonolinga	45	54	2–4 (3; 2–4)	99
Bafia	42	40	2–4 (3; 2–4)	82
Ntui	87	82	2–4 (3; 2–4)	169
Yabassi	35	36	2–4 (3; 2–4)	71
Total, *n* (%)	209 (49.6)	212 (50.4)	2–4 (3; 2–4)	421 (100)

*IQR* interquartile range

### Prevalence and intensity of *O. volvulus* infection among children < 5 years

A total of 318 children were examined for onchocerciasis in the Bafia, Ntui and Yabassi Health Districts. The prevalence of *O. volvulus* microfilaridermia was 6.6% (95% *CI*: 4.3–9.9). The Bafia Health District exhibited the highest infection rate (12.2%; 95% *CI*: 6.8–21.2) compared to the other Health Districts (*χ*^2^: 6.79; df: 2; *P*-value: 0.0335) (Table 2). The infection rate was also similar between males (8.7%; 95% CI 5.1–14.1) and females (4.8%; 95% *CI*: 2.0–9.1) (*χ*^2^: 2.20; df: 1; *P-*value: 0.1362) (Table 2).

**Table 2:** Prevalence and intensity of *O. volvulus* infection according to gender and Health District

Variables	No. children examined	No. children with Skin mf	Infection rate (95% *CI*)
Health Districts			
Bafia	82	10	12.2% (6.8–21.2%)
Ntui	169	6	3.6% (1.4–7.7%)
Yabassi	67	5	7.5% (2.9–16.7%)
Gender			
Males	162	14	8.7% (5.1–14.1%)
Females	156	7	4.8% (2–9.1%)
Overall	318	21	6.6% (4.3–9.9%)

*No.* number of; *CI* confidence interval; *mf* microfilariae

**Fig. 1:**
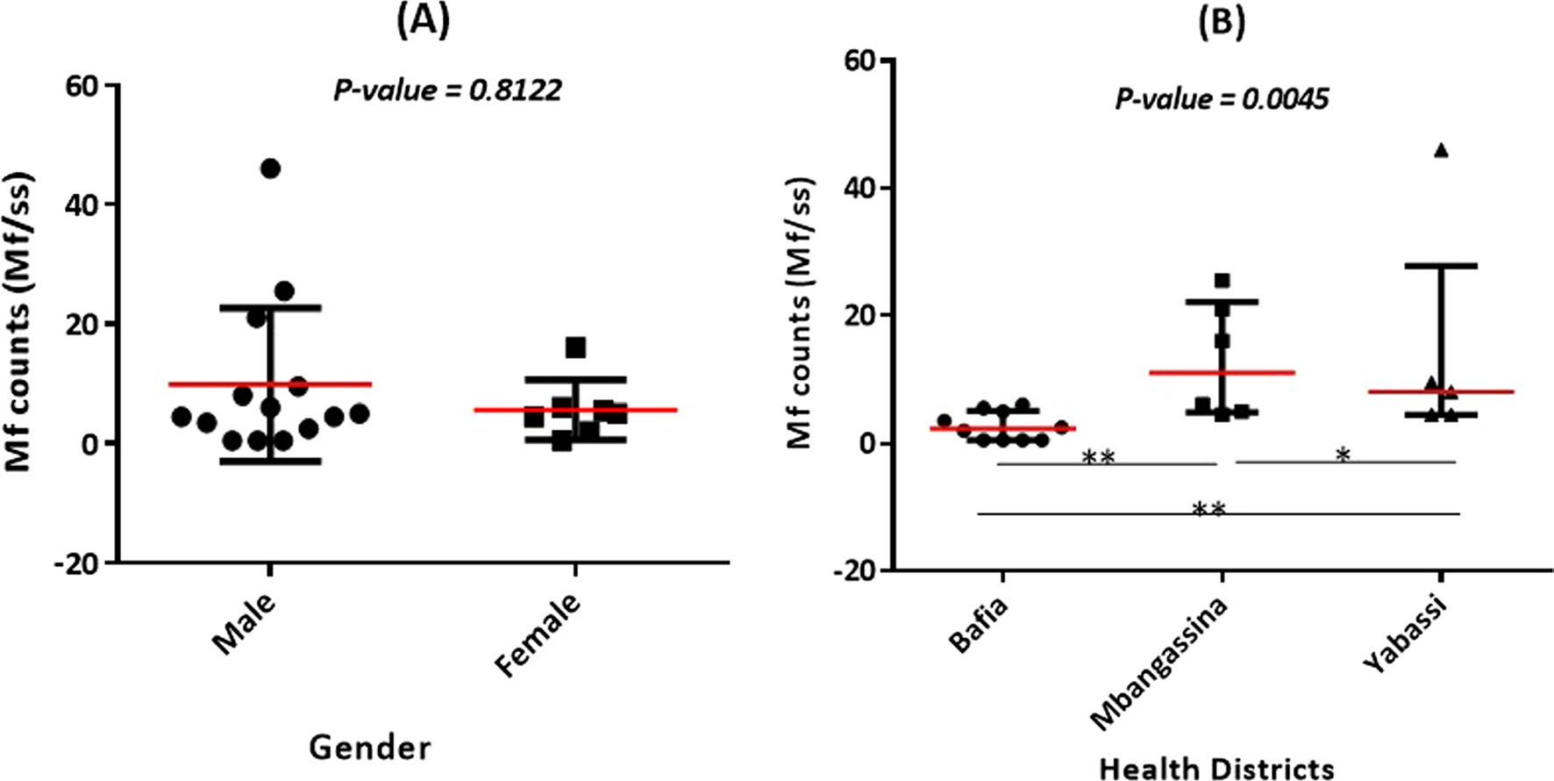
Intensity of onchocerciasis infection in the study population. **A** Comparison of *O. volvulus mf* counts between boys and girls; **B** comparison of *O. volvulus mf* counts between the different Health Districts. mf: Microfilariae. *Non significant difference. **Significant difference

Onchocerciasis intensity of infection for microfilaremic individuals ranged from 0.5 to 46 microfilariae per skin snip (Median: 5; IQR: 2.25–8.75 mf/ss). The intensity of infection was similar between girls and boys (Mann Whitney U: 45.5; *P*-value: 0.8122) (Fig. 1A). However, high heterogeneity was observed in the intensity of infection between Health Districts, the mean mf counts being significantly different between the Health Districts (*χ*^2^: 9.327; *P*-value: 0.0045) (Fig. 1B). The Dunn’s Multiple comparison revealed that the intensity of infection was significantly higher in Bafia and Ntui Health District, compared to the Yabassi Health District (mean rank difference: − 8.633) (Fig. 1B).

### Prevalence and intensity of STH infection among children < 5 years

A total of 250 children successfully provided stool samples in the two Health Districts (Akonolinga and Ntui) surveyed for STH, of whom 24 (9.6%; 95% *CI*: 6.5–13.9) harbored at least one of the two main STH species (*A. lumbricoides* and *T. trichiura*) (Table 3). Infected children were found only in the Akonolinga Health District; the infection rate was 29.6% (95% *CI*: 28.8–40.4). *A. lumbricoides* was the most prevalent STH species (7.6%) among infected children, and one (0.4%) child was co-infected by both STH species. No significant difference was found in the infection rate between girls (10.2%) and boys (8.9%) (*χ*^2^: 0.12; df: 1; *P-*value: 0.729).

**Table 3:** Prevalence and intensity of STH infection according to gender and Health District

Variables	No. children examined	No. children harboring *A. lumbricoides* (%)	No. children harboring *T. Trichiura* (%)	No. children harboring at least one STH	Infection rate (95% *CI*)
Health Districts					
Akonolinga	81	19 (23.5)	6 (7.4)	24	29.6% (28.8–40.4%)
Ntui	169	0 (0.0)	0 (0.0)	0	0.0% (0.0–2.7%)
Gender					
Males	123	9 (7.3)	2 (1.6)	11	8.9% (4.9–15.5%)
Females	127	10 (7.9)	4 (3.1)	13	10.2% (5.9–16.9%)
Overall	250	19 (7.6)	6 (2.4)	24	9.6% (6.5–13.9%)

*No.* number of; *CI* confidence interval; *STH* soil-transmitted helminth

**Fig. 2:**
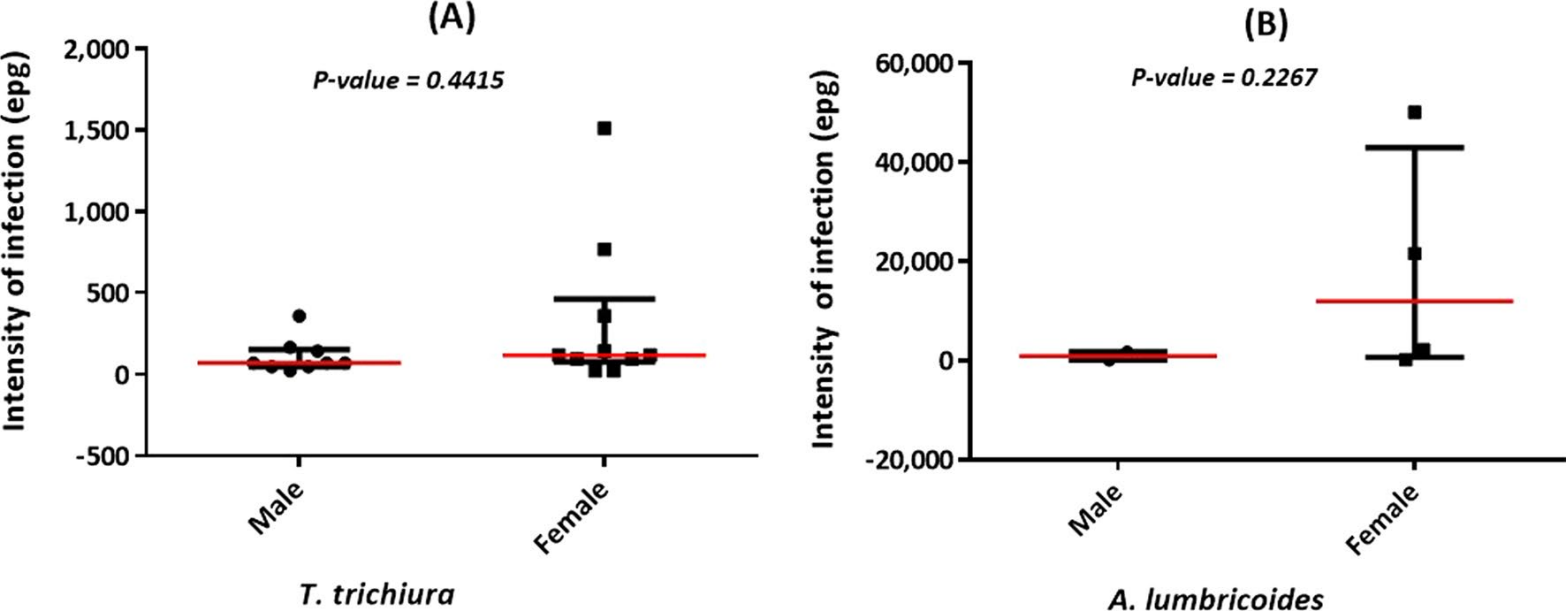
Intensity of STH infections in the study population. **A** Comparison of *T. trichiura* intensity between boys and girls; **B** comparison of *A. lumbricoides* intensity between boys and girls. epg: number of eggs per gram of stool

The intensity of infection ranged from light to heavy infection for *A. lumbricoides* (192 to 50,088 epg; Median: 1,992; IQR: 210–28,704), and was light for all the infected children for *T. trichiura* (24 to 1,512 epg; Median: 96; IQR: 48–168). Likewise infection rate, the intensity of infection was similar between girls and boys, both for *A. lumbricoides* (Mann Whitney U: 1; *P*-value: 0.2667) (Fig. 2A) and *T. trichiura* (Mann Whitney U: 32; *P*-value: 0.3012) (Fig. 2B).

## Discussion

The WHO 2030 target for onchocerciasis and STH are to interrupt the transmission in 31% of the endemic countries, and achieve and maintain the elimination as public health concern, respectively [6, 18, 19]. However, the strategies used to control/eliminate those infections generally left behind individuals aged < 5 years. To bring more evidence supporting the need of initiating or intensifying intervention in this age class, we are reporting the magnitude of onchocerciasis and STH in children aged 2–4 years in different settings with different levels of endemicity to both diseases in Cameroon, and discuss their impacts in the prospect of elimination.

In this study, although the global proportion of STH was quite low (9.6%), the proportion of infected children was 29.6% (95%* CI*: 28.8–40.4) in the Akonolinga Health District, with the intensity of infection ranging from light to heavy. These high rates and intensities of STH infection among pre-SAC in this area might be explained by the fact that very high infection rates have been previously found both among school-aged children and adults in the Akonolinga Health District [22], and as such the likelihood to have younger individuals (pre-SAC) infected as well is high. Additionally, the ongoing deworming activity in the area follows the school-based model, leaving somehow behind or neglected pre-SAC who are not typically reached during school-based deworming campaigns. As a consequence, those children are at high risk of developing STH-related morbidity such as malnutrition and anemia, diminished physical fitness, growth retardation and delayed intellectual development [32, 33] since the intensity of infection was found to be light or heavy for *A. lumbricoides*. In addition, if left untreated, those children might serve as parasites reservoir and source of re-infestation for their older counterparts who are receiving PC at schools, thus maintaining a vicious cycle of transmission and hindrance to elimination efforts. This finding adds to growing evidence that school-based deworming alone is not sufficient to interrupt STH transmission as previously demonstrated [22, 28]. This raise the need of implementation or intensification of control approaches, such as the deworming through Child Health Days (recommended by the WHO) [34], Child and Maternal Health and Nutrition Action Week (SASNIM) as it is the case in Cameroon, or the reconsideration of community-based model for an optimal coordination of STH control, at least in areas of high transmission [35, 36].

The rates and intensities of onchocerciasis infection recorded in this study are almost similar to figures recorded among individuals aged ≥ 5 years old [25, 37], suggesting that in these highly endemic areas children under 5 years have almost similar risk of infection as their older counterparts. Besides this, the prevalence of onchocerciasis was significantly lower in Mbangassina (Ntui Health District) compared to Bafia and Yabassi Health Districts. This is not surprising since the three areas exhibit different patterns of onchocerciasis transmission. Indeed, Bafia and Yabassi Health Districts are meso-endemic foci where the transmission is still unexpectedly high despite more than 20 years of MDA [25], while in the Ntui Health District, onchocerciasis prevalence has dropped from 26.3% in 2009 to 8.3% in 2020 [38, 39]. This suggests that the risk of onchocerciasis infection in children < 5 years is positively associated with the level of endemicity of onchocerciasis, and further suggest that these heavily infected children might contribute to the transmission, thus disrupting onchocerciasis elimination prospects due to continued transmission. Also, this might be one of the factors underlying the persistence of onchocerciasis in those communities, despite more than 20 years of control efforts [25]. In addition, the high intensity of infection found in these children is of great concern at a public health perspective since such high burden of *O. volvulus* parasites might lead to serious health and socio-economic consequences such as OAE and nodding syndrome [15–17]. Indeed, it was demonstrated that early childhood infection with *O. volvulus* is associated with an increased risk of developing either seizures or epilepsy, with a strong dose-response relationship with the intensity of infection [14]. In fact, the prepatent period of *O. volvulus* infection is estimated to 7 to 12 months [40]. If children are exposed to infection quite early in their life, they will already have fully reproductive parasites at 4 years; this might further explain the high prevalence of onchocerciasis associated epilepsy in the Mbam valley [15, 16, 41]. This quite high prevalence and intensity of onchocerciasis infection among children aged 2 to 4 years, and the potential health and socio-economic consequences, raise the need of intervention for this age class, and it appears worth to conduct trials investigating the IVM dose that can safely clear *O. volvulus* mf in this age class, or alternatively to develop a pediatric formulation of IVM.

The main limitation of this study is the small sample size at the level of Health Districts (71‒169 children aged less than 5 years of age), consecutive to the difficulty of collecting skin biopsies (painful procedure) or stool samples from this specific age class. Although this small sample size may reduce the power of statistical tests, the objective of this study was to investigate to what extent children excluded from preventive chemotherapies were infected with onchocerciasis and STH in order to prompt decisions for the control of these parasitic infections among these age groups. Further investigations with larger sample size and broader breadth of coverage across targeted age class (including children aged less than 2 years old) might be useful to better estimate the burden of onchocerciasis and STH among children excluded from IVM- or MEB/ALB- based PC, though the present study already provide important enough data for decision making.

## Conclusions

Relatively high rates and intensities of STH and onchocerciasis infections have been found among children aged 2 to 4 years old in this study. Beside important health and socio-economic consequences that can arise as these children are infected quite early but left untreated, this high endemicity may also constitute a serious obstacle to the prospects of elimination of those diseases. There is therefore an urgent need to reconsider the role played by these children in the control of onchocerciasis and STH, so as to indeed end the neglect and move towards the sustainable development goals leaving no one behind.

## Data Availability

The data supporting the conclusions of this article are included within the article. Raw data can be obtained from corresponding authors upon reasonable request.

## Abbreviations

ALB: Albendazole
CDTI: Community-directed treatment with IVM
*CI*: Confidence interval
HD: Health Districts
IQR: Interquartile range
IVM: Ivermectin
MDA: Mass drug administration
MEB: Mebendazole
mf: Microfilariae
OAE: Onchocerciasis-associated epilepsy
PC: Preventive chemotherapy
Pre-SAC: Pre-school-aged children
SAC: School-aged children
SASNIM: Semaine d'Actions de Santé et de Nutrition Infantile et Maternelle
ss: Skin snip
STH: Soil-transmitted helminthiases
WHO: World Health Organization
