# Correction to: High infection rates for onchocerciasis and soil-transmitted helminthiasis in children under five not receiving preventive chemotherapy: a bottleneck to elimination

**DOI:** 10.1186/s40249-022-00984-y

**Published:** 2022-06-06

**Authors:** Hugues C. Nana-Djeunga, Linda Djune-Yemeli, André Domche, Cyrille Donfo-Azafack, Arnauld Efon-Ekangouo, Cédric Lenou-Nanga, Narcisse Nzune-Toche, Yves Aubin Balog, Jean Gabin Bopda, Stève Mbickmen-Tchana, Thirumalaisamy P. Velavan, Véronique Penlap-Beng, Francine Ntoumi, Joseph Kamgno

**Affiliations:** 1 Centre for Research on Filariasis and Other Tropical Diseases (CRFilMT), Yaoundé, Cameroon; 2 Molecular Diagnosis Research Group, Biotechnology Centre, University of Yaoundé I, Yaoundé, Cameroon; 3 Department of Biochemistry, Faculty of Science, University of Yaoundé I, Yaoundé, Cameroon; 4 Parasitology and Ecology Laboratory, Department of Animal Biology and Physiology, Faculty of Science, University of Yaoundé I, Yaoundé, Cameroon; 5 Institute for Tropical Medicine, University of Tübingen, Tübingen, Germany; 6 Fondation Congolaise Pour la Recherche Médicale (FCRM), CG-BZV, Brazzaville, Republic of the Congo; 7 Faculty of Science and Technology, Marien Ngouabi University, Brazzaville, Republic of the Congo; 8 Department of Public Health, Faculty of Medicine and Biomedical Sciences, University of Yaoundé I, Yaoundé, Cameroon

## Corection to: Infectious Diseases of Poverty (2022) 11:1–8 10.1186/s40249-022-00973-1

Following publication of the original article [1], it was found that one co-author’s first name and family name were interchanged by mistake, the correct name should be Thirumalaisamy P. Velavan.

The original paper has been [1] updated.

## References

[1] Nana-Djeunga HC, Djune-Yemeli L, Domche A, Donfo-Azafack C, Efon-Ekangouo A, Lenou-Nanga C, Nzune-Toche N, Balog YA, Bopda JG, Mbickmen-Tchana S, Velavan TP, Penlap-Beng V, Ntoumi F, Kamgno J (2022). High infection rates for onchocerciasis and soil-transmitted helminthiasis in children under five not receiving preventive chemotherapy: a bottleneck to elimination. Infect Dis Poverty.

